# Associations between Cognitive and Affective Responses to Tobacco Advertisements and Tobacco Use Incidence: A Four-Year Prospective Study among Adolescent Boys

**DOI:** 10.3390/ijerph182111666

**Published:** 2021-11-06

**Authors:** Brittney Keller-Hamilton, Hayley Curran, Elise M. Stevens, Michael D. Slater, Bo Lu, Megan E. Roberts, Amy K. Ferketich

**Affiliations:** 1 Division of Medical Oncology, Department of Internal Medicine, The Ohio State University College of Medicine, Columbus, OH 43214, USA; 2 Center for Tobacco Research, The Ohio State University Comprehensive Cancer Center, Columbus, OH 43214, USA; 3 Division of Epidemiology, The Ohio State University College of Public Health, Columbus, OH 43210, USA; curran.144@osu.edu (H.C.); ferketich.1@osu.edu (A.K.F.); 4 Division of Preventive and Behavioral Medicine, Department of Population and Quantitative Health Sciences, University of Massachusetts Medical School, Worcester, MA 01605, USA; elise.stevens@umassmed.edu; 5 School of Communication, The Ohio State University College of Arts and Sciences, Columbus, OH 43210, USA; slater.59@osu.edu; 6 Division of Biostatistics, The Ohio State University College of Public Health, Columbus, OH 43210, USA; lu.232@osu.edu; 7 Division of Health Behavior and Health Promotion, The Ohio State University College of Public Health, Columbus, OH 43210, USA; roberts.1558@osu.edu

**Keywords:** advertising, electronic cigarettes, cigarettes, smokeless tobacco, youth, longitudinal study

## Abstract

Exposure to tobacco advertisements is associated with initiation of tobacco use among youth. The mechanisms underlying this association are less clear. We estimated longitudinal associations between youths’ cognitive and affective responses to advertisements for cigarettes, e-cigarettes, and smokeless tobacco (SLT) and initiation of these products. *N* = 1220 Ohio-residing boys of ages 11–16 were recruited into a cohort in 2015 and 2016. Participants completed surveys every six months for four years. Surveys assessed cognitive and affective responses to tobacco advertisements (which included health warnings) and tobacco use after an advertisement viewing activity. We used mixed-effects Poisson regression models with robust standard errors to estimate risk of initiating use of each tobacco product according to participants’ cognitive (i.e., memorability of health risks) and affective (i.e., likability of advertisement) responses to advertisements for that product. No associations between affective responses to advertisements and tobacco use outcomes were detected in adjusted models. However, finding health risks memorable was associated with reduced risk of ever smoking initiation (aRR = 0.57; 95% CI: 0.34, 0.95) and a reduced risk of ever SLT initiation that approached statistical significance (aRR = 0.61; 95% CI: 0.36, 1.05). Measures to increase saliency of health risks on cigarette and SLT advertisements might reduce use among youth.

## 1. Introduction

Over the past decade, the prevalence of cigarette smoking and smokeless tobacco (SLT) use has declined among youth, reaching an estimated 3.3% and 2.3%, respectively, of youth in 2020 [1,2]. Conversely, the prevalence of electronic cigarette (e-cigarette) use has generally increased over the past decade and peaked in 2019, when an estimated 20% of youth reported current use of e-cigarettes [3]. In 2020, current use of e-cigarettes decreased but remained high at 13.1% of youth [1]. Increased exposure to tobacco marketing is consistently associated with cigarette smoking, SLT use, and e-cigarette use among youth [4,5,6,7,8]. Youths’ receptivity to tobacco advertisements (operationalized as their ability to name a favorite brand and owning or being willing to own a tobacco promotional item) is also associated with use of these products [9,10,11].

According to the McGuire communication–persuasion matrix, receptivity to an advertisement is based on a hierarchy of items, including exposure, cognitive response, and affective response [12,13,14]. Cognitive responses require analytical and conscious processing [15,16] and can be measured by asking people to report their thoughts or what they remember about an advertisement, such as health risks. Affective responses to advertisements reflect emotional processing [17] and can be measured by asking participants how much they liked an advertisement or how the advertisement made them feel [18]. Together with message comprehension, cognitive and affective responses contribute to one’s perceptions of the product and ultimately one’s decisions to purchase or use the product [12,13,14].

Although this theoretical framework exists, our understanding of the associations between cognitive and affective responses to tobacco advertisements and tobacco use outcomes among youth is limited. A large body of research has investigated the associations between advertisement exposures, advertisement receptivity, and tobacco use [4,5,6,7,8,9,10,11]. Beyond receptivity, much less is understood about associations between youths’ other cognitive and affective responses toward advertisements and tobacco use. This gap is notable because youths’ cognitive and affective responses—especially the counterarguments they generate [19]—to tobacco advertisements could provide useful targets for public health measures. Through prevention interventions, youths’ tobacco advertisement exposures and reactions to advertisements are modifiable risk factors for initiation of tobacco use.

Our goal was to assess the associations between cognitive and affective responses to tobacco advertisements, operationalized as memorability of health risks and likeability of the advertisement, respectively, and prospective initiation of cigarette smoking, e-cigarette use, and SLT use. We hypothesized that youth who found health risks less memorable and advertisements more likeable would have increased risk of becoming ever or current tobacco users over the four-year study.

## 2. Materials and Methods

### 2.1. Setting and Participants

Male youth (*N* = 1220) were recruited into a longitudinal study between January 2015 and June 2016. Girls were not recruited because one of our aims was to evaluate predictors of SLT use, which is rare among girls [1] and would have required a substantially larger sample size. Eligible boys were aged 11 to 16 years and lived in either urban Franklin County, Ohio, or one of nine Appalachian Ohio counties. Exclusion criteria included inability to read or speak English and vision, hearing, or cognitive impairments that would affect data collection. Participants were recruited using a combination of probability address-based sampling (*N* = 991) and convenience sampling (*N* = 229). Convenience sampling methods were used due to slow recruitment and less income and racial/ethnic diversity using probability sample alone. Additional details about sampling and recruitment are provided elsewhere [20].

### 2.2. Procedures

#### 2.2.1. Baseline

Trained interviewers administered a questionnaire to male youth participants during an in-person interview. Sensitive items (e.g., tobacco use, other substance use, and peer tobacco use) were administered via audio computer-assisted self-interviewing (ACASI). Remaining items (e.g., exposure to advertisements in magazines, cognitive and affective responses to advertisements) were interviewer-administered. Parents or guardians of the male youth completed a self-administered survey to report indicators of family socioeconomic status and household tobacco use.

An advertisement viewing activity was completed during the interviewer-administered portion of the visit [20,21,22]. During this activity, the interviewer displayed five print advertisements for eight seconds each, then assessed male youths’ cognitive and affective responses using qualitative and quantitative measures (described below) after each advertisement was viewed. Each participant viewed advertisements for three tobacco products (cigarettes, e-cigarettes, and SLT) and two beverage products (alcohol and non-alcoholic beverages). Advertisements were randomly ordered and randomly selected from a larger stimulus set of advertisements that appeared in recent print issues of *People Magazine*, *Sports Illustrated*, *Popular Science*, *ESPN Magazine*, and *Rolling Stone*, magazines with relatively high youth readership. The size of the stimulus set ranged from 13 to 30 advertisements for each product at each time point. All advertisements were one page. Advertisements that were primarily sponsorships for concerts or sporting events were excluded, as were advertisements that were overtly sexual. After the interview, interviewers debriefed participants about the advertisement viewing activity. They told participants that advertisements do not show the negative consequences of using the advertised product, including cancer, heart disease, and motor vehicle crashes.

#### 2.2.2. Follow-Up Surveys

Follow-up interviews were conducted every six months for four years. The two-year follow-up was conducted in-person, but all other follow-ups were conducted via telephone. The two-year follow-up used the same procedures as baseline, including for the advertisement viewing activity. The follow-up surveys conducted via telephone were shorter, predominantly assessed tobacco use, and did not include the advertisement viewing activity. The two-year follow-up in-person surveys were completed between January 2017 and August 2018 (73.7% retention) and the four-year follow-up phone surveys were completed between January 2019 and June 2020 (62.3% retention).

### 2.3. Measures

#### 2.3.1. Predictor Variables

Cognitive and affective responses to tobacco advertisements were the predictors of interest. Responses were assessed at baseline and the two-year follow-up, and thus were modeled as time-varying predictors.

Cognitive responses were measured with an open-ended item: “Name one thing about the ad that you particularly noticed or remembered.” Participants spoke their response aloud to the interviewer, who transcribed their responses verbatim. Participants were permitted to list multiple memorable components of the advertisement if they wanted, and in these cases each component was coded. Responses were coded by independent coders with good interrater reliability following procedures reported previously (range of Krippendorff’s alpha values: 0.78 to 1.00) [21,22]. Briefly, coders underwent extensive training to establish acceptable interrater reliability for all codes. Then, responses were coded in batches with intermittent reliability assessments to ensure inter-rater reliability remained acceptable. Responses that were difficult to code were reviewed as a group to reach consensus for a final code. Responses that indicated participants found the health warning, other health risks, or the addiction potential of the tobacco product most memorable were combined to create a variable representing that health risks were memorable for the participant after viewing the tobacco advertisement (coded yes/no). We did so as cognitive responses that have the most impact on persuasion, according to the Elaboration Likelihood Model (ELM) [19], are those that counterargue the persuasive theme of the message. Statements regarding health risks of tobacco products represent such counterarguments and thus the cognitive responses of greatest interest regarding the potential impact of advertisement on future behavior.

Affective response was derived from three close-ended items asking participants to rate (0–10) how enjoyable, likeable, and appealing they found the advertisement [17]. Values were averaged to derive one mean affective response score for the advertisement (Cronbach’s alphas > 0.91), with higher values representing a more positive response. Due to mean affective responses being strongly right skewed, categorical variables were created: 0 vs. >0 mean affective response [20].

#### 2.3.2. Outcome Variables

Initiation of cigarettes, e-cigarettes, and SLT by the two-year and four-year follow-ups were the outcomes of interest. At the two-year follow-up, participants were classified as having initiated ever use of each product if they were never users at baseline and reported having tried the product at least once at or before their two-year follow-up interview. At the four-year follow-up, participants were classified as having initiated ever use of each product if they had never used the product at the two-year follow-up but reported using the product at least once at or before their four-year follow-up survey. Similar coding was used to establish initiation of current use of each product, although past 30 day use of each product was used to determine initiation at the two- and four-year follow-ups.

#### 2.3.3. Confounding Variables

Several covariates were conceptually identified as confounders of the association between cognitive and affective responses to tobacco advertisements and initiation of tobacco use. Confounding variables included age in years, race and ethnicity (White non-Hispanic; Black non-Hispanic; and Hispanic, other race, or multiple races), region (Appalachian vs. non-Appalachian Ohio), parental education (<Bachelor’s degree vs. Bachelor’s degree or higher), household tobacco use (does not live with vs. lives with an adult who uses tobacco), peer tobacco use (none vs. a few or more friends use tobacco), recalled exposure to magazine advertisements in general (never or rarely vs. sometimes or more often), reported grade point average (GPA), sensation seeking mean (a measure of impulsivity; Cronbach’s alphas at each wave >0.7), ever alcohol use, and ever use of other tobacco products. For time-varying confounders (i.e., age, peer tobacco use, recalled exposure to magazine advertisements, GPA, sensation seeking, ever alcohol use, and ever tobacco use), values from baseline and the two-year follow-up were included in models assessing two-year and four-year tobacco initiation, respectively.

### 2.4. Statistical Analysis

We used all available follow-up data to assess initiation of cigarettes, e-cigarettes, and SLT. Baseline advertisement responses were used to predict initiation at any time during the first two years of follow-up, and two-year follow-up advertisement responses were used to predict initiation at any time during the last two years of follow-up. In other words, tobacco use outcomes of interest in longitudinal models occurred at the two- and four-year follow-up surveys. When covariate values changed over time, they were treated as time-varying covariates in models as described above.

Descriptive statistics were calculated for all variables to characterize the sample at baseline, the two-year follow-up, and the four-year follow-up. Next, separate mixed-effects Poisson regression models with robust standard errors were run to estimate associations between predictor variables and outcome variables; Poisson regression was used to facilitate estimation of risk ratios [23]. Cognitive and affective responses to product-specific advertisements were linked to initiation for the same tobacco product. For example, responses to SLT advertisements were predictors in models assessing SLT initiation. Initial models included a fixed effect for wave and random intercepts. Due to very small counts of non-White participants who used SLT, and the fact that race is a strong correlate of SLT use and likely a strong confounder if left uncontrolled [1], all SLT outcome models were restricted to non-Hispanic White participants.

Prior to running fully adjusted analyses, we conducted model assumption checks for linearity and multicollinearity. This testing resulted in decisions to standardize GPA and sensation seeking scores in all models, include a quadratic term for GPA in cigarette ever use incidence models, and include quadratic terms for sensation seeking in e-cigarette and SLT current use incidence models. Finally, separate fully adjusted mixed-effects Poisson regression models with robust standard errors were used to estimate risk of ever/current initiation of cigarettes, e-cigarettes, and SLT associated with cognitive and affective responses to advertisements for each respective product. Predictor variables, confounders, and an indicator for wave were included as fixed effects, and random intercepts were also included. Analyses were completed using Stata/SE version 17 (College Station, TX, USA).

## 3. Results

### 3.1. Participant Characteristics

At baseline, male youth participants were 14.1 years old on average, 75.8% were White non-Hispanic, 42.0% lived in Ohio Appalachia, and 31.8% lived with an adult tobacco user. Approximately half of male youth participants had a parent or guardian with a Bachelor’s degree or higher (Table 1). On average, participants were 16.0 years old at the two-year follow-up and 17.3 years old on average at the four-year follow-up, reflecting greater attrition of older participants as the study progressed. Distributions of demographic characteristics were largely similar across each wave, although a larger proportion of participants had a parent or guardian with a Bachelor’s degree or higher after baseline. As expected, descriptively, a higher proportion of participants reported ever use of alcohol or tobacco products as the study progressed. E-cigarettes were the most-used tobacco product at each wave, and e-cigarettes also had the highest incidence of any tobacco product at each wave (Table 2).

### 3.2. Descriptive Assessment of Cognitive and Affective Responses to Tobacco Advertisements

For cognitive responses, the proportion of male youth participants who found health risks memorable after viewing a cigarette advertisement was stable from baseline to the two-year follow-up (20.1% vs. 20.5%, respectively; Table 1). For e-cigarette advertisements, the proportion of male youth participants who found health risks memorable decreased from 14.7% at baseline to 8.2% at the two-year follow-up. For SLT advertisements, the proportion who found health risks memorable increased from 46.2% at baseline to 51.2% at the two-year follow-up.

Descriptively, affective responses to cigarette advertisements appeared to be stable from baseline to the two-year follow-up (55.1% >0 vs. 57.3% >0, respectively; Table 1). Affective responses appeared to become more positive over time toward e-cigarette (53.2% >0 at baseline vs. 60.4% >0 at the two-year follow-up) and SLT advertisements (27.5% >0 at baseline vs. 53.7% at the two-year follow-up).

### 3.3. Associations between Cognitive and Affective Responses to Tobacco Advertisements and Tobacco Use Incidence

In models that only adjusted for wave, having a mean affective response >0 was associated with increased risk of initiating ever and current cigarette smoking and e-cigarette use among our participants (Table 3). Finding health risks memorable after viewing cigarette advertisements was associated with reduced risk of ever cigarette smoking initiation. The association between finding health risks memorable after viewing SLT advertisements and reduced risk of SLT initiation approached statistical significance (*p* = 0.06). No other associations were statistically significant.

Adjusting for confounders attenuated the associations between affective responses to tobacco advertisements and initiation of cigarette smoking and e-cigarette use to non-significance among our participants. However, the association between finding health risks memorable after viewing cigarette advertisements and reduced risk of cigarette smoking initiation remained statistically significant. The association between finding health risks memorable on SLT advertisements and reduced risk of SLT initiation again approached statistical significance (*p* = 0.08).

## 4. Discussion

We found that liking cigarette and e-cigarette advertisements was associated with increased risk of initiating ever cigarette and e-cigarette use, respectively, in unadjusted models. Additionally, finding health risks memorable after viewing cigarette advertisements was associated with reduced risk of cigarette smoking incidence among our participants. In adjusted models, however, associations between liking cigarette and e-cigarette advertisements and ever use were attenuated to non-significance, but the association between finding health risks of smoking memorable and reduced risk of cigarette smoking initiation remained. Consistent but nearly-significant associations were detected between finding health risks memorable on SLT advertisements and reduced risk of initiating ever SLT use among our participants.

Exposure to tobacco advertisements has consistently been associated with increased risk of tobacco use among youth [4,5,6,7,8]. Therefore, understanding the mechanisms underlying this association would provide useful targets for developing prevention-oriented policies and interventions. From prior work, it appears that increased exposure to tobacco advertisements is associated with holding more positive beliefs and attitudes toward tobacco use [24,25]. However, we are unaware of research that has prospectively linked advertisement beliefs and attitudes to tobacco use outcomes, and thus the best way to use these findings for prevention efforts is unclear. Our investigation into cognitive and affective responses to tobacco advertisements found that thinking about health risks after viewing cigarette advertisements is a protective cognitive response. Thus, policies and interventions to increase the saliency of health risks on cigarette advertisements could be useful to further decrease the prevalence of youth smoking. Further investigation into why similar associations were not found for e-cigarette advertisements is needed. Our null findings could be due in part to using e-cigarette advertisements from before the FDA-mandated large health warnings on e-cigarette advertisements [26]. Although the e-cigarette advertisements in our stimulus set included voluntary health warnings [27], these were often small and difficult to read. It is also possible that normative beliefs about e-cigarettes among adolescents contributed to differences in their attitudes toward e-cigarette advertisements [28].

The associations between cognitive responses to SLT advertisements and SLT use longitudinally, which approached statistical significance in our sample, are also a target for further investigation, as prevalence of SLT use among males in Appalachian Ohio remains high [29]. It is possible that we would have detected a statistically significant effect with a larger sample size given the magnitude of our point estimates. It is also possible that the reasons youth start to use SLT are different from the reasons youth start to use other tobacco products, particularly in our sample where many boys who started using SLT lived in Appalachian Ohio. In some Appalachian families, for example, SLT use is viewed as a rite of passage or way to emulate male family members [30]. Altogether, memorability of health risks in SLT advertisements might play a relatively smaller role in initiation than is the case for cigarettes.

We did not identify a statistically significant association between affective responses to tobacco advertisements and tobacco use outcomes longitudinally in the fully adjusted models in our sample. This finding might suggest that prevention efforts focused on youths’ affective responses to tobacco advertisements should not be prioritized. However, it is also possible that our measure of affective response, which was strongly right skewed with roughly half of participants having an average score of 0, failed to adequately represent the construct. Research using more comprehensive measures of affective responses might be more informative.

One limitation of this study was that participants were randomly assigned to view different advertisements. Thus, it is likely that some of the variability between participants’ responses is due to viewing different advertisements rather than differences in how participants cognitively or affectively respond to tobacco advertisements in general. Relatedly, we cannot tease apart whether the descriptive differences we observed in cognitive and affective responses to e-cigarette and SLT advertisements over time are due to temporal differences in the advertisements themselves, changes in how adolescents process advertisements as they age, or other prevailing norms related to using these products that could contribute to advertisement processing. Another limitation was that we relied solely on self-report data to capture participants’ tobacco use and cognitive and affective responses to tobacco advertisements. Although we used ACASI to assess tobacco use, it is possible that participants underreported tobacco use. Advertisement responses were reported to an interviewer, who was often much older than the participant and typically a woman. Complementing these self-report measures with psychophysiological measures (e.g., heart rate, recognition tasks, and eye-tracking) would provide information about cognitive and affective processing in real time that are not directly accessible through self-report [31,32,33]. Additionally, because health warning requirements differed substantially between cigarette, e-cigarette, and SLT advertisements while our study was in the field, it is difficult to directly compare associations between finding health risks memorable and tobacco use outcomes across different products because these associations might be confounded by the size and content of the health warning. Finally, all study participants were under the age of 21 at the time of their final survey. The city of Columbus (located in Franklin county) began enforcing a new Tobacco 21 policy in October 2017 (increasing the minimum legal sales age for tobacco to 21) and Ohio began enforcing a state Tobacco 21 policy in October 2019 [34], which occurred mid-way through our four-year survey. It is possible that Tobacco 21 affected tobacco use incidence.

## 5. Conclusions

We evaluated associations between cognitive and affective responses to tobacco advertisements and incident tobacco use over four years of follow-up among male youth. Overall, we identified that associations between affective responses to cigarette and e-cigarette advertisements and initiation of those products were attenuated after controlling for confounding factors. However, the association between thinking about health risks after viewing a cigarette advertisement and reduced risk of cigarette smoking initiation remained statistically significant. In fact, our youth participants who thought about health risks after viewing a cigarette advertisement were over 40% less likely to have tried smoking a cigarette during follow-up. Our findings provide direction for policies and interventions aimed at reducing cigarette smoking among youth, as well as direction for future research to more comprehensively assess how youth process tobacco advertisements and how this processing is associated with initiation of tobacco use.

## Figures and Tables

**Table 1:** Characteristics of male youth participants at baseline and two- and four-year follow-ups.

	Baseline *N* = 1220	Two-Year Follow-Up *N* = 899	Four-Year Follow-Up *N* = 760
**Age, *mean* (*SD*)**	14.1 (1.6)	16.0 (1.6)	17.3 (1.6)
**Race and ethnicity, *n* (%)**			
White non-Hispanic	925 (75.8)	699 (77.8)	596 (78.4)
Black non-Hispanic	154 (12.6)	98 (10.9)	77 (10.1)
Hispanic, other race, or multiple race	141 (11.6)	102 (11.4)	87 (11.5)
**Region, *n* (%)**			
Appalachia Ohio	512 (42.0)	348 (38.7)	290 (38.2)
Non-Appalachian urban Ohio	708 (58.0)	551 (61.3)	470 (61.8)
**Parental Education, *n* (%)**			
<Bachelor’s degree	565 (46.3)	351 (39.0)	299 (39.3)
Bachelor’s degree or higher	655 (53.7)	548 (61.0)	461 (60.7)
**Household tobacco use, *n* (%)**			
Does not live with an adult tobacco user	832 (68.2)	661 (73.5)	543 (71.5)
Lives with an adult tobacco user	388 (31.8)	238 (26.5)	217 (28.6)
**Peer tobacco use, *n* (%)**			
None	684 (63.2)	386 (47.1)	267 (36.1)
A few or more	398 (36.8)	434 (52.9)	473 (63.9)
**Magazine advertisement exposure**			
Never or rarely	1053 (86.6)	714 (92.0)	-
Sometimes or often	163 (13.4)	62 (8.0)	-
**GPA, *mean* (*SD*)**	3.29 (0.8)	3.34 (0.6)	-
**Sensation seeking, *mean* (*SD*)**	2.87 (0.9)	2.94 (0.9)	-
**Ever alcohol use, *n* (%)**			
No	994 (84.9)	680 (77.4)	463 (61.1)
Yes	177 (15.1)	199 (22.6)	295 (38.9)
**Ever used any tobacco product, *n* (%)**			
No	995 (81.6)	654 (73.1)	464 (61.1)
Yes	225 (18.4)	241 (26.9)	296 (39.0)
**Mean affective response to cigarette advertisement, *n* (%)**			
0	547 (45.0)	331 (42.7)	-
>0	670 (55.1)	444 (57.3)	-
**Mean affective response to e-cigarette advertisement, *n* (%)**			
0	571 (46.8)	307 (39.6)	-
>0	648 (53.2)	468 (60.4)	-
**Mean affective response to SLT advertisement, *n* (%)**			
0	590 (72.5)	359 (46.3)	-
>0	225 (27.5)	416 (53.7)	-
**Memorable health risks—cigarette advertisement, *n* (%)**			
No	964 (79.9)	612 (79.5)	-
Yes	242 (20.1)	158 (20.5)	-
**Memorable health risks—e-cigarette advertisement, *n* (%)**			
No	1041 (85.3)	713 (91.8)	-
Yes	179 (14.7)	64 (8.2)	-
**Memorable health risks—SLT advertisement, *n* (%)**			
No	656 (53.8)	377 (48.8)	-
Yes	563 (46.2)	395 (51.2)	-

Note: Counts might not sum to the total sample size due to item nonresponse. Percentages might not sum to 100 due to rounding. Variables without descriptive statistics at the four-year follow-up were not assessed at that wave. Abbreviations: GPA = grade point average and SLT = smokeless tobacco.

**Table 2:** Prevalence of tobacco use at baseline and incidence at two- and four-year follow-ups.

	Baseline *N* = 1220	Two-Year Follow-Up *N* = 899		Four-Year Follow-Up *N* = 760	
	*N* (%) Prevalent Use	*N* at Risk ^a^	*N* (%) Initiated	*N* at Risk ^a^	*N* (%) Initiated
**Ever use**					
Cigarettes	103 (8.4)	848	56 (6.6)	595	62 (10.4)
E-cigarettes	123 (10.1)	824	79 (9.6)	560	68 (12.1)
SLT	89 (7.3)	851	36 (4.2)	608	35 (5.8)
**Current use**					
Cigarettes	25 (2.1)	889	30 (3.4)	640	28 (4.4)
E-cigarettes	29 (2.4)	879	48 (5.5)	618	51 (8.3)
SLT	28 (2.3)	883	17 (1.9)	648	23 (3.6)

Abbreviations: E-cigarette = electronic cigarette and SLT = smokeless tobacco. ^a^ The count of participants at risk for tobacco use refers to the number of participants who completed the follow-up survey and were not ever/current tobacco users prior to that survey.

**Table 3:** Associations between cognitive and affective responses to tobacco advertisements and tobacco use incidence over four years of follow-up ^a^.

	Cigarette Incidence RR (95% CI)				E-Cigarette Incidence RR (95% CI)				SLT Incidence ^b^ RR (95% CI)			
	Ever Use	*p*-Value	Current Use	*p*-Value	Ever Use	*p*-Value	Current Use	*p*-Value	Ever Use	*p*-Value	Current Use	*p*-Value
**Model 1 ^c^**												
Mean affective response > 0 ^d^	1.54 (1.04, 2.27)	0.03	1.98 (1.09, 3.61)	0.03	1.64 (1.16, 2.31)	<0.01	1.78 (1.13, 2.80)	0.01	0.97 (0.53, 1.79)	0.91	0.66 (0.31, 1.41)	0.29
Health risks were memorable ^e^	0.54 (0.31, 0.95)	0.03	1.16 (0.61, 2.19)	0.65	1.15 (0.73, 1.83)	0.55	1.27 (0.72, 2.25)	0.40	0.60 (0.35, 1.02)	0.06	1.28 (0.66, 2.50)	0.46
**Model 2 ^f^**												
Mean affective response > 0 ^d^	1.37 (0.90, 2.10)	0.15	1.66 (0.85, 3.24)	0.14	1.07 (0.73, 1.56)	0.73	0.92 (0.58, 1.44)	0.70	0.70 (0.40, 1.23)	0.22	0.63 (0.29, 1.41)	0.26
Health risks were memorable ^e^	0.57 (0.34, 0.95)	0.03	1.25 (0.68, 2.32)	0.47	1.06 (0.63, 1.79)	0.82	1.07 (0.58, 2.00)	0.83	0.61 (0.36, 1.05)	0.08	1.19 (0.61, 2.33)	0.62

Abbreviations: E-cigarette = electronic cigarette; SLT = smokeless tobacco; RR = risk ratio; CI = confidence interval; ^a^ Adolescent male participants viewed randomly-selected and randomly-ordered advertisements for cigarettes, e-cigarettes, and SLT at baseline and the two-year follow-up. Tobacco use was assessed every six months from baseline to the four-year follow-up. Mixed-effects Poisson regression models with robust standard errors were used to estimate associations between cognitive and affective responses to cigarette, e-cigarette, and SLT advertisements and respective tobacco use outcomes. ^b^ Due to small counts of non-White participants who initiated SLT over the study period, SLT models were restricted to non-Hispanic White male youth. ^c^ Model 1 controlled for wave. ^d^ Participants reported how enjoyable, likeable, and appealing they found each advertisement (response scale: 0–10). Responses were averaged (Cronbach’s alphas > 0.91) and dichotomized to 0 vs. > 0 due to right-skewed distributions. ^e^ Participants reported what they found “most memorable” about the tobacco advertisement. Responses that the warning, other health risks, or addictiveness of the tobacco product was the most memorable part of the advertisement were combined. ^f^ Model 2 controlled for wave, tobacco retailer visits, frequency of seeing tobacco advertisements in magazines, age, race and ethnicity, region, parental education, GPA, use of other tobacco products, alcohol use, living with an adult tobacco user, sensation seeking, and peer tobacco use. For time-varying covariates, values from the same wave as advertisement response were used.

## Data Availability

The data presented in this study are available upon reasonable request to the corresponding author.
